# The Impact of “Omic” and Imaging Technologies on Assessing the Host Immune Response to Biodefence Agents

**DOI:** 10.1155/2014/237043

**Published:** 2014-09-16

**Authors:** Julia A. Tree, Helen Flick-Smith, Michael J. Elmore, Caroline A. Rowland

**Affiliations:** ^1^Biodefence and PreClinical Evaluation Group, Public Health England (PHE), Porton Down, Salisbury, Wiltshire, SP5 3NU, UK; ^2^Biomedical Sciences Department, Defence Science and Technology Laboratory (Dstl), Porton Down, Salisbury, Wiltshire, SP4 0JQ, UK

## Abstract

Understanding the interactions between host and pathogen is important for the development and assessment of medical countermeasures to infectious agents, including potential biodefence pathogens such as *Bacillus anthracis*, *Ebola virus*, and *Francisella tularensis*. This review focuses on technological advances which allow this interaction to be studied in much greater detail. Namely, the use of “omic” technologies (next generation sequencing, DNA, and protein microarrays) for dissecting the underlying host response to infection at the molecular level; optical imaging techniques (flow cytometry and fluorescence microscopy) for assessing cellular responses to infection; and biophotonic imaging for visualising the infectious disease process. All of these technologies hold great promise for important breakthroughs in the rational development of vaccines and therapeutics for biodefence agents.

## 1. Introduction

Understanding host-pathogen interactions is important for the development and assessment of medical countermeasures to infectious agents. The advent of new imaging and “omic” technologies has provided the ability to follow these interactions from whole animal to cellular and molecular levels, enabling a greater understanding of the mechanisms involved; this facilitates the development and refinement of new and existing vaccines and therapeutics. For example, advances in bioimaging provide a noninvasive means of identifying the internal systemic spread of infection in animal models and the impact of a prophylaxis or a therapy on the disease process. This can be combined with the analysis of responses at a cellular level using flow cytometry and microscopy techniques. The use of microarrays has also enhanced our understanding of the host response to infection and provides supportive information to help elucidate the innate and adaptive immune mechanisms essential for protection against pathogens, as well as the virulence mechanisms deployed by the pathogen. Although in its infancy, next generation sequencing also holds great potential for defining host-pathogen interactions. This review will assess the impact of these technologies on the ability to assess the host response and how this has been applied to help progress the development of vaccines and immunotherapies against biodefence agents described in the Centers for Disease Control and prevention (CDC) Select Agent list (http://www.selectagents.gov/). Biodefence agents are dangerous pathogens that require high levels of biocontainment and are relatively less-studied (compared with the majority of public health pathogens) and cases are relatively rare. Therefore, studies to test the efficacy of therapeutics in a healthy population from an endemic area are often not feasible and the use of animal models is essential. This review focuses on the use of these new techniques to help us understand host responses in animal models as well as humans. In this context, both “omic” and imaging technologies hold great promise for important breakthroughs in the rational development of vaccines and therapies.

## 2. “Omic” Technologies

Traditionally, many immunological studies have focused on examining single immune parameters, such as cytokines, using techniques like ELISA and ELISpot. This approach does not highlight interconnecting pathways that control the immune response when the host encounters an infectious agent. With the emergence of transcriptomic technologies, such as microarray and next-generation sequencing, thousands of parameters of the immune system can be measured at the same time at a genome-wide scale. This allows a systematic, unbiased approach to understand how transcript changes correlate with diverse states of the immune system [1]. This section aims to review the use of microarrays and next-generation sequencing in relation to defining the host response against biodefence agents, vaccines, and therapies.

### 2.1. Microarrays

#### 2.1.1. DNA Microarrays

A DNA microarray consists of a solid surface, usually a glass microscope slide onto which DNA molecules (probes), in picomolar concentrations, are chemically bonded. The purpose of a microarray is to detect the presence and abundance of labelled nucleic acids (targets) in a biological sample, which will hybridise to the DNA on the array. The level of binding between a probe and its target is quantified by measuring the fluorescence emitted by the hybridized targets when scanned. In the majority of microarray experiments the labelled nucleic acids are derived from the mRNA of a sample or tissue, and so the microarray measures gene expression [2].

Most microarrays are prepared so that they cover the whole genome of a species; however, in the absence of a fully sequenced organism, researchers have used smaller focused arrays designed from publically available gene sequences [3, 4]. Alternatively, whole genome microarrays have been used from related animals to predict immune profiles [5] or new arrays have been constructed using cross-species hybridisation bioinformatics to create probes to unsequenced genes [6]. These kinds of approaches are currently being superseded by the use of next-generation sequence analysis which can generate new sequence information rapidly and accurately. On occasion this information has been used to build new microarray platforms; a successful example of this has been applied to the ferret model of influenza [7]. Today, DNA microarrays have been constructed for studying gene expression changes in a number of different species including the mouse, rat, cow, dog, cat, chicken, horse, pig, rabbit, sheep, guinea pig, ferret, chimpanzee, marmoset, rhesus, and cynomolgus macaque.

DNA microarrays have revolutionized our understanding of the host gene expression changes in response to infection with various pathogens. This information has largely been obtained from* in vitro *infection experiments. Primary cells taken from naïve human volunteers [8–15] or continuous cell lines [16–22] have been infected and incubated with a pathogen for different time periods (ranging from 1 to 48 hours) and host gene signatures generated (Table 1). Microarray studies performed in this way provide insights into the cellular response following infection with, for instance, Monkeypox virus. Alkhalil et al. (2010) showed that many genes (89.08%) in MK2 cells underwent downregulation by 1.5-fold changes or more [21] following infection with Monkeypox virus. Bourquain et al. (2013) also found major unresponsiveness of HeLa cells after exposure [16]. Rubins et al. (2011) concluded, from studies on different human cell types, that Monkeypox virus selectively inhibited the expression of genes with critical roles in cell-signalling pathways that activate innate immune responses (such as TNF-*α*, IL-1 *α* and *β*, CCL5, and IL-6) [14]. Thus it would appear that Monkeypox virus downregulates or silences genes so that the host is less responsive to infection.

DNA microarray analysis has been used to improve our understanding of the host response following exposure to the bacterium [18], spores [9], edema toxin [17], and lethal toxin [8] of* Bacillus anthracis*, the causative agent of anthrax. Studies on human peripheral monocytes revealed that anthrax lethal toxin targets multiple normal immune-regulatory pathways that would be expected to protect the host against anthrax infection. They hypothesised that the increase in RGS14 levels and decrease in CCR5, along with IL-1R2, impairs monocyte function and facilitates bacterial survival [8].

Despite the ready availability of DNA microarrays for use with different animal species, relatively few* in vivo* transcription studies have been published using models of infection with biodefence infectious agents compared with public health pathogens such as tuberculosis (TB) or human immunodeficiency virus (HIV). Using the mouse model, gene signatures have been determined in different organs following infection with* Burkholderia pseudomallei* [23, 24], Venezuelan equine encephalitis virus (VEEV) [25, 26], and* Francisella tularensis* [27–30]. Very recently a bovine model has been used for investigating host mRNA expression changes to* Brucella melitensis* by examining the infected Peyer's patch from a calf ligated ileal loop. This study showed that the early infectious process of* Brucella* was primarily accomplished by compromising the mucosal immune barrier and subverting critical immune response mechanisms [31].

Some microarray studies have been performed using nonhuman primates (NHPs) infected with Ebola virus [32] and Variola virus [5]. In studies at Public Health England the mRNA profiles of NHPs infected with Monkeypox virus and* B. anthracis* are currently underway (personal communication, Karen Kempsell). There is scope for many more informative microarray studies to be performed in various animal models of biodefence agents.

#### 2.1.2. Protein Microarrays

Protein microarray is a more recent technology, providing a platform for high-throughput proteomics. Construction is similar to DNA microarrays, except that the immobilised species is a protein or a peptide, and the array aims to represent partially or wholly the entire proteome [52]. Two methods of protein generation are used: (1) the “standard” method where the gene for each protein is amplified, cloned, produced in an* in vitro* expression system (typically in* Escherichia coli*), and printed directly onto glass slides [46]; (2) an alternative method where the encoding DNA is printed onto the slide and expressed* in situ* at the time required (NAPPA, nucleic acid programmable array) [53].

One of the most powerful applications of protein microarrays is in the study of the humoral immune response to infection. Arrays have been used to assess host antibody profiles (or “immunosignature”) in response to infection with* B. melitensis* [34, 35],* B. pseudomallei* [33, 54], Vaccinia/Variola virus [47], Monkeypox virus [39], and* Coxiella burnetii* [36–38] (Table 1). Studies on* C. burnetii*, the etiological agent of Q-fever, have helped to identify new diagnostic antigens [36, 38, 45]. Seven* C. burnetii* proteins (GroEL, YbgF, RplL, Mip, OmpH, Com1, and Dnak) were identified (from protein arrays studies) and then fabricated on a small array and tested with sera from patients with other diseases (Rickettsial spotted fever,* Legionella* pneumonia, or* Streptococcal* pneumonia) as well as Q-fever, in order to develop a diagnostic assay. The selected antigens demonstrated moderate specificity for recognizing Q-fever in patient sera [38]. The use of protein microarrays has also aided the identification of different IgG and IgM profiles for differentiating acute and chronic Q-fever [37] and a proof-of-concept diagnostic assay (immunostrip) to distinguish the two disease states [37]. In addition to identifying antigens for diagnostic tools, antibody profiling, using protein arrays, also provides candidate antigens for subunit vaccine development [37].

#### 2.1.3. Use of Microarrays for the Evaluation of Vaccines and Therapies

Microarray technology has been used to help understand the cell-mediated and humoral immune responses following infection with infectious agents; furthermore it has also improved our understanding of the mechanism of action of therapeutics and biodefence vaccines. For instance a transcriptomic approach, using DNA microarrays, was used to assess the host response to treatment with therapeutic agents (rNAPc2 or rhAPC) designed to block the coagulation pathway during Ebola virus infection in NHPs [32]. Coagulation abnormalities in Ebola hemorrhagic fever have been previously reported [55] suggesting that blocking the development of coagulopathies during Ebola virus infection might limit pathogenesis. Microarray analysis showed that the overall circulating immune response in NHPs was similar both in the presence and absence of coagulation inhibitors; however, the profiles of the surviving NHPs in the treated groups clustered together [32]. Only small numbers (2/8 and 2/11) of animals survived in each treatment group but the study did reveal that several differentially expressed genes correlated with survival, namely, chemokine ligand 8 (CCL8/MCP-2) and coagulation-associated genes TFPI and PDPN [32]. Further work is clearly needed in this area as these genes may provide possible targets for early-stage diagnostics or future therapeutics.

A limited number of studies have been performed using DNA microarrays to understand the underlying protective mechanisms of licensed or novel biodefence vaccines. DNA arrays have been used to examine the immunostimulatory properties of CpG motifs [40–42] which when used as an adjuvant have been shown to significantly prolong the protection induced by anthrax vaccine adsorbed (AVA) [56, 57]. Recently, Paranavitana and colleagues examined the transcriptional profile of human volunteers who had received the live vaccine strain (LVS), an attenuated strain of* F. tularensis* [43]. PBMCs from individuals were restimulated with LVS* in vitro* and memory responses were evaluated. The microarray results revealed that both dendritic cells and macrophages played significant roles in antigen presentation. Significantly differentially expressed genes including IL-15, GM-CSF, IL-9, and IL-10 as well as genes associated with T-cell, B-cell, and natural killer cell activities were identified. Paranavitana et al. concluded that the manipulation of the dendritic cell maturation pathway, with stimuli to promote efficient antigen presentation, may be a way forward for future vaccine development against* Francisella* [43].

The antibody profile evoked by smallpox vaccines has been examined in detail following the development of a Vaccinia proteome microarray by Davies et al. in 2005 [46]. Since then, the immunosignature evoked by three different vaccines (Acam2000, Dryvax, and MVA) in the mouse, rabbit, macaque, prairie dog, and humans have been defined [47–50]. Follow-up studies using protein arrays involved examining the sera from more than 2000 smallpox-vaccinated humans. Six dominant antigens were identified comprising 3 membrane and 3 nonmembrane antigens from the intracellular mature virion [51]. These antigens were then evaluated in an ELISA format with sera from MVA and DryVax vaccinees. Overall, these ELISAs should aid in monitoring the human immune response to MVA in both vaccinia naïve and previously vaccinated individuals, thus assisting with vaccine development in the future.

Protein arrays have also been used to examine the immunosignature of mice vaccinated with killed* F. tularensis* LVS adjuvanted with immune stimulating complexes (ISCOMS) and CpG [44]. Similarly, protein arrays were used to assess the immunosignature of Q-Vax (Q-fever) vaccine [45]. Both studies identified protective proteins which should aid the design of new or improved vaccines.

Advances in “omic” technology have also assisted with the identification of candidate T-cell antigens. An ORFeome flexible cloning approach was developed by Jing and colleagues whilst analysing the CD4 T-cell response to vaccinia virus using PBMCs from Smallpox vaccinated individuals in 2009 [58]. This method has since been used to identify candidate T-cell antigens for herpes simplex virus type 1 (HSV-1) [59]. This could be applied to the identification of T-cell antigens for Biodefence vaccines.

### 2.2. Next Generation Sequencing

Next generation sequencing (NGS; also known as high-throughput, short-read, or deep sequencing) has revolutionised sequence-based analyses over the last decade. The underlying principle is that it uses micro-/nanotechnologies to run millions of parallel sequencing reactions, generating millions or billions of bases per run (which is up to 6 logs greater than the output using the Sanger method). Read lengths are typically comparatively short, a result of which is that any particular base is sequenced many times (known as coverage or read depth). There are a number of competing platforms, with Illumina, ABI SOLiD, Roche 454, and Ion Torrent technologies being widely used, each having different characteristics with regard to average read length, total bases sequenced per run, and cost-per-base [60–62]. Other more specialized NGS technologies are PacBio Single Molecule Real Time (SMRT) and Oxford Nanopore [63, 64].

NGS technology can be applied to both DNA (DNA-seq), and RNA (after conversion to cDNA-RNA-seq). RNA-seq analysis aims to identify the transcriptome (the complete set of transcripts of the cell, which includes mRNA, noncoding RNAs, and small RNAs). RNA-seq is increasingly being used as an alternative to microarray as a method of measuring gene expression [65, 66] and uses the sequence read depth of the RNA species as a measure of the absolute level in the sample. The two methods have a high degree of correspondence [67, 68], and similar analytical statistical techniques can be used, although data preprocessing and sample normalization require different bioinformatic techniques [69]. RNA-seq is reported to have significant advantages over microarray, such as less bias/variation, lower background signal, and a larger dynamic range (up to 100-fold greater). Importantly, it does not depend on prior knowledge of a reference transcriptome and therefore can lead to discovery of previously unknown RNA species and of “edited” RNA species such as splice variants [70]. However, certain disadvantages do exist, such as nonuniform read coverage, inability to detect a rare transcript (unless high read depth is obtained), and discrepancy in read depth or library sizes between samples [65].

#### 2.2.1. Use of RNA-Seq to Study Host Response to Pathogens

Upon infection of a host with a pathogen, changes in the expression of both organisms occur. These are usually investigated separately due to the low pathogen:host transcript ratio (up to 200-fold); thus enrichment of the pathogen transcripts is often required [71]. Pathogen expression profiling examples include analysis of the* F. tularensis* transcriptome during infection of mouse macrophages [72] and sRNA expression of* Yersinia pestis* grown* in vitro* and in the mouse lung [73].

RNA-seq has been used to investigate the host response to different virulent strains of* B. melitensis* in mouse peritoneal macrophages [74]. Compared with previous microarray studies, deep sequencing provided a more sensitive and comprehensive unbiased coverage of the host transcriptome, with many alternative and novel transcripts being discovered. In particular, it was shown that a live attenuated vaccine strain (M5-90) had a reduced ability to avoid phagosome-lysosome fusion and activate MAPK pathways when compared with the virulent strain M28. This may account for the difference in the ability of the two strains to survive in the host [74]. A second study examining the microRNA (miRNA) profile of RAW264.7 cells in response to* B. melitensis* infection also used a high throughput sequencing approach [75]. Zheng and colleagues concluded that* Brucella* may establish a chronic infection by regulating the host miRNA profile [75].

The human host response to Dengue virus infection has also been reported using RNA-Seq [76]. A significant amount of previously uncharacterised gene isoforms and alternative transcripts over a range of pathways were shown, and particularly there was a greater number of host differentially regulated transcripts upon infection by an attenuated DENV strain than by the wild-type, suggesting that there may be a previously uncharacterised innate immune response which is largely evaded in wild-type strains [76].

#### 2.2.2. Dual RNA-Seq

Ideally it would be preferable to monitor the gene expression profiles of the pathogen and host simultaneously. This “dual RNA-seq” approach is technically and bioinformatically more challenging [77, 78] but may well become the established method. However, recent examples do exist for the simultaneous profiling of the host and viral [79] or bacterial [29, 80] species. For instance, Walter and colleagues exposed mice to virulent* F. tularensis* and discovered that, while acute infection at four hours was associated with marked suppression of multiple aspects of the innate immune response (relative to other pathogens examined), a subset of immune-related transcripts was uniquely induced by* Francisella*. They also showed that a classical inflammatory response was activated in the lungs of mice, 24 hours after infection and this simultaneously correlated with a dramatic change in bacterial gene expression patterns [29]. These results should help to identify potential virulence factors which target host inflammatory pathways, in the future.

Dual RNA-seq has also been used to evaluate the immune response following smallpox vaccination. PBMCs taken from Dryvax vaccinated individuals were either stimulated with or without live Vaccinia virus for 8 hours [81]. Results showed detection of all annotated Vaccinia genes, with those genes classified as “early” in the viral life cycle expressed at significantly higher levels. On the host side numerous innate genes and pathways were activated upon vaccinia infection. A number of chemokines, cytokines, interferons, and macrophage-associated genes exhibited downregulation upon infection whilst there was an upregulation of histones, IFN*β*, IFN*γ*, and heat shock proteins [81].

#### 2.2.3. Other Uses of NGS Sequencing

*T and B Cell Repertoire Diversity.* The immunological repertoire is a term defining the collection of surface-expressed B-cell (BCRs) and T-cell receptors (TCRs). Receptor diversity is generated dynamically by sequence rearrangement of specific loci in the germline genome, leading to a vast diversity of differing clones [82]. Classically, studies on the immune repertoire have used techniques that either provide a limited description or sample a limited number of sequences (e.g., CDR3 spectratyping, targeted sequencing [83]). The high-throughput nature of NGS technology allows simultaneous analysis of potentially the entire immune repertoire in a single experiment (using DNA-Seq or RNA-Seq) [84–86]. Recent applications have included studying the changes in the antibody responses to Dengue virus infection [87] and Influenza vaccination [88] and there is clearly scope to apply this technique to monitor the immune repertoire in response to other infectious diseases, vaccines, or therapeutics.

## 3. Optical Imaging of Host-Pathogen Interactions

Imaging infection using optical sources relies on the detection of specific targets using fluorescence or bioluminescence. Fluorescent light is emitted with a characteristic emission spectrum following excitation at specific wavelengths. Fluorescent molecules may be used to tag specific molecules of interest. Very often the molecule of interest will be an antibody which in turn will be directed to specific targets (e.g., surface receptors on host cells). Alternatively, endogenous proteins can be made to fluoresce, for example, in genetically modified animals or pathogens, or fluorescent dyes can be used to label pathogens or cells. Bioluminescence is produced by the reaction of a luciferase enzyme with its substrate and requires energy and oxygen to occur. Unlike fluorescence imaging, where the signal is still detectable for some hours after the host has died, bioluminescent imaging requires living cells. This section aims to review how our understanding of biodefence pathogens, vaccines, and immunotherapies and their interactions with the host has been greatly aided by imaging techniques such as flow cytometry, fluorescence microscopy and real time* in vivo* biophotonic imaging.

### 3.1. Flow Cytometry

This technique is routinely used as an important tool for assessing cellular responses to infection and vaccination in both human patients and animal models of infection. It is used for cellular phenotyping and functional assays including fluorescence-based proliferation assays. Bead-based assays are also available to assess levels of soluble factors including cytokines in samples from* in vitro* and* ex vivo* tissues. Intracellular cytokine responses can also be measured by intracellular staining, with fluorochrome labelled antibodies, to determine cellular phenotypes generated following vaccination or therapeutic treatment with specific antigens. Antibodies for specific cell targets are generally available for a number of animal species particularly the mouse and rat, but cell target ranges are limited for less commonly used species such as the marmoset, which in turn can limit the analysis of cellular responses in these models. In order to understand host-responses, fluorescently labelled pathogen-specific antibodies allow the presence of intracellular bacteria to be identified within host cells [89]. Alternatively bacteria expressing fluorescent molecules, for example, green fluorescent protein (GFP) [90], m-cherry red [91] or other fluorescent markers may be used. In combination with specific antibody staining of host cells, fluorescent labelling of pathogens has allowed the location of pathogens within host cells to be identified in both* in vitro* and* in vivo* infection studies using flow cytometry and immunofluorescence microscopy.

#### 3.1.1. Pathogenesis and Assessment of Immunotherapies

Flow cytometry has highlighted a key role for various cell types in murine infection models of* Y. pestis*, the causative agent of plague. The importance of neutrophils in respiratory* Y. pestis* was demonstrated in two early studies [92, 93]. Furthermore, flow cytometry was used to identify the target host cells of* Y. pestis* in a murine pneumonic infection model where alveolar macrophages (CD11c^+^CD11b^+^F4/80^+^) were identified as the initial cell type to uptake the bacterium followed by neutrophils [94]. An additional study investigating intratracheal inoculation of* Y. pestis* showed the interaction of this bacterium with CD11c^+^DEC205^+^CD11b- cells in the airways and lung. Depletion of this cell type suggested an important role for it in the initial replication and dissemination of* Y. pestis* from the lung [95]. It is speculated that the difference in cellular tropism of* Y. pestis* described in these respiratory studies maybe due to the difference between aerosol and intratracheal dosing and differing strains of the organism. In an intradermal model of infection,* Y. pestis* was also found to reduce the activation of inflammatory cells, particularly neutrophils, at the site of infection. Using bacterial mutants, the* Y. pestis* virulence plasmid pYV was shown to be involved in this evasion of early inflammatory responses in the skin [96].

Neutrophil inflammatory responses have been characterised following infection in mouse models with both* B. pseudomallei* [97] and* Burkholderia mallei* [98] by flow cytometry where neutrophils were found to be crucial for protection in both respiratory and intraperitoneal forms of these related infections. The role of neutrophils in* Burkholderia* infection in the murine host also aligns with human* ex vivo* studies where phagocytosis and apoptosis of* B. pseudomallei* by human blood neutrophils were impaired in neutrophils from diabetic patients. This impaired neutrophil function may contribute to the increased susceptibility to* Burkholderia* infection observed in diabetic patients [99].

Using flow cytometry to understand host-pathogen interactions has the potential to enable an association between host immune markers with protective effects following treatment with therapeutics and vaccines. A number of studies have assessed the immune response to immunotherapeutic approaches for treatment of infection to further understand potentially protective immune responses in* in vivo* models of infection using immunostimulants including CpG motifs [100]. One study using phosphoantigens as an immunotherapy in a marmoset model of* B. pseudomallei* infection [101] showed that, although there was no effect on survival, strong cell-mediated immune responses were detected which could inform future treatment strategies. In another study, decreased bacterial numbers and increased survival to a novel immunotherapeutic strategy using Acai polysaccharides against pulmonary* F. tularensis* or* B. pseudomallei* infection* in vivo* [102] were associated with IFN-*γ* production by NK cells in the lung.

Flow cytometry bead-based assays have the potential to aid our understanding of potential protective mechanisms of novel immunotherapeutics by assessing cytokine responses. These assays have been used in studies investigating the effects of IFN-*γ* therapy in mice during* B. pseudomallei* infection [103] and to understand changes occurring as a result of treatment with an HMGB1-antibody antibiotic combination therapy [104]. This study showed that treated mice had significantly higher IFN-*γ* levels which correlated with survival. They have also been used to further our understanding of the protective immune responses involved following a combination of preexposure vaccination and postexposure CpG immunotherapy against* B. pseudomallei* infection* in vivo* [105] where both intranasal and intraperitoneal vaccinations with 2D2 attenuated vaccine strain were found to generate antigen-specific IFN-*γ* CD4^+^ T cell responses. A greater pulmonary T cell response was observed following vaccination via the intranasal route which corresponded with increased protection against pulmonary infection.

#### 3.1.2. Vaccine Studies

Immune responses elicited* in vivo* following immunization and* ex vivo* cellular restimulation with specific antigens from vaccinated or infected animals have been used to determine specific, memory-type responses to vaccines. This has broadened our understanding of a number of vaccination strategies for biodefence pathogens including live vaccines for* B. pseudomallei* [106] and* F. tularensis* [107], novel live vaccine strategies against* F. tularensis* infection [108], and heat-killed vaccines to* B. mallei* [109]. Potential immune correlates of protection have also been identified in studies by comparing profiles of lymphocyte populations following vaccination (prior to infection) and their responses during infection with Monkeypox virus [110]. In depth assessment of T-cell signatures in vaccines or individuals with naturally acquired* F. tularensis* infection suggested that these signatures could be used to identify protective correlates of immunity to* F. tularensis* [111]. Additionally, the identification of putative vaccine candidates [112] and the longevity of immune responses to vaccines have also been greatly aided by flow cytometry in follow-on studies of* F. tularensis* live vaccine strain (LVS) vaccination [111, 113]. Immune responses to vaccination have also been used in preclinical animal models (murine and primate) and in Phase I clinical trials to assess host responses to a recombinant plague vaccine. Although a number of memory cell phenotypes were investigated, flow cytometry lacked the sensitivity to detect changes in immune profiles between vaccinated and placebo groups [114]. Understanding immune responses generated by novel vaccines can facilitate the rational development of vaccines which induce the most appropriate immune responses to protect against infection, for example, by engineering the known protective F1-antigen against* Y. pestis* to include B-cell and T-cell epitopes [115].

#### 3.1.3. Other Uses of Flow Cytometric Techniques

In addition to examining lymphocyte responses, the role of antigen-presenting cells in generating protective immunity during vaccination to* B. pseudomallei* (e.g., dendritic cells (DCs) [116]) and* F. tularensis* LVS [107] has also been aided by the use of flow cytometry techniques. The use of a GFP strain (BP82-GFP) of an intradermally delivered live, attenuated* B. pseudomallei* vaccine [117] and specific cell staining demonstrated that the most efficient cell type at uptake and transport of bacteria to the draining lymph node was the neutrophil.

Fluorescent-activated cell sorting and cDNA technologies have recently been used together to generate antigen-specific monoclonal antibodies [118]. The overall aim of this is the provision of antibody treatments for infectious diseases and an example of this has already been applied to emerging coronavirus species including severe acute respiratory syndrome (SARS) [119]. This technology may well be used for developing therapeutics for biodefence agents in the future. Other potential applications of flow cytometry in biosecurity research, outside the area of investigation of host-pathogen interactions, are reviewed in Marrone, 2009 [120].

### 3.2. Fluorescence Microscopy

A number of recent advances in fluorescent microscopy techniques such as confocal microscopy, intravital 2-photon microscopy, dynamic live cell imaging, and super resolution microscopy have been used to interrogate host-pathogen interactions, based on detection of specific fluorescent signals to provide detailed images of pathogens colocalised with or within host cells. Fluorescence microscopy is also being investigated for its utility in the diagnosis of infections including* B. pseudomallei*. In comparison with flow cytometry, fluorescence microscopy can provide a much more detailed assessment of how pathogens interact with individual host cells. These studies are aided through the use of a range of fluorescent dyes for both the pathogen and cellular structures/organelles including DNA dyes such as DRAQ5 or DAPI which are important for nuclear identification.

#### 3.2.1. *In Vitro* Infection Models

Fluorescence microscopy has enabled us to understand the intracellular nature and niches of pathogens and their ability to evade immune pathways to enable their survival within host cells. For example, identifying the lysosomal escape mechanism of* F. tularensis* into the cytosol provided an understanding of its ability to survive within host macrophages [121]. Fluorescence microscopy has also been used to investigate the role of complement in uptake of* F. tularensis* into cells [122] and the immune evasion of this pathway by* F. tularensis* [123]. It has also been used to understand the effect of modulators on macrophage function and phagosomal escape of* F. tularensis* LVS [124] and the interaction of* B. mallei* with macrophages* in vitro* to assess which bacterial components are important in the pathogenesis of disease [125]. Understanding the effects of* F. tularensis* LVS strains and their altered interactions with host cells [126] has potential implications for future licensing of these vaccines. Our understanding of the interaction of* F. tularensis* and other bacteria with host cells and its intracellular nature has contributed to development of treatment regimens including delivery of antibiotic therapies suitable for treating intracellular infection. Fluorescence microscopy also has the potential to elucidate host pathway targets which could be therapeutically manipulated to prevent evasion of the host response by bacteria and aid in intracellular clearance. For example, the interaction of* F. tularensis* and* B. pseudomallei* with pathways of autophagic digestion has been assessed using fluorescence microscopy in* in vitro* studies. The interaction of* B. pseudomallei* with autophagosomes using GFP-LC3-expressing RAW 264.7 cells and fluorescently labelled bacteria (both wildtype and mutants) elucidated mechanisms of evasion of the autophagic pathway by* B. pseudomallei* [127].* F. tularensis* was also found to utilize one autophagic pathway for its survival [111]. Further studies identified potential new therapeutic compounds to target the autophagic pathway [128, 129]. It was demonstrated that inducing alternative autophagic pathways using the novel inducer AR-12 reduced bacterial growth in* F. tularensis* infection [112]. Confocal scanning microscopy has enhanced our understanding of the interaction of* C. burnetii* with host-cells and intracellular vacuoles [130]. The dynamics between vacuoles, lysosomes, and the processes which regulate actin dynamics in formation of vacuoles, including GTPases and associated proteins, have been investigated in detail using* C. burnetii* mutants [107]. This has led to identification of host targets with the potential for therapeutic targeting. Confocal microscopy has also been utilised to develop a high content imaging assay to understand the formation of multinucleated giant cells (MNGCs) during* B. pseudomallei* infection which is a unique mechanism used by* Burkholderia* spp. and is thought to allow spread of the infection without detection by the immune system [131]. This quantitative method allowed the effect of bacterial mutants, thought to subvert the formation of MNGCs, to be assessed. Importantly, this method was also used to investigate the effect of small molecule inhibitors on MNGC formation and thus has the potential to be used as a screening tool for novel therapeutics. Confocal microscopy has also been used to investigate the interaction of viral pathogens including filoviruses such as Ebola virus with host cells [132, 133] and by mapping the interaction between the viral proteins polymerase L and its cofactor VP-35. In this model* in vitro* system, immunofluorescence analysis demonstrated that this interaction was disrupted by mutants containing the VP-35 binding site which led to reduced Ebola virus replication, thus identifying a potential target for development as a novel antiviral therapy [134].

#### 3.2.2. *In Vivo* Infection Models

*F. tularensis* was detected by phase-contrast and fluorescence microscopy using* in situ* hybridization [135] and an m-cherry red strain of* B. melitensis* identified bacteria associated with host cells in tissue sections from* in vivo* infection models [91]. This study showed the* in situ* colocalisation of* B. melitensis* with a number of different cellular phenotypes within granulomas in the spleen and liver. Understanding these immune-pathological lesions using complex immunohistochemistry and fluorescent bacterial strains has the potential to allow identification of new treatments for bacteria which so effectively evade and manipulate host responses to enable their survival. The binding of monoclonal antibody therapies to the* Y. pestis* bacterium using immunofluorescence demonstrated the specificity of potential therapies for bubonic plague. This prior testing of monoclonal antibody therapies was important to determine the specificity of any protection observed in* in vivo* studies [136]. Intravital microscopy, which adds an additional parameter of time to microscopy studies, was used to identify the rapidity of the neutrophil response* in situ* during intradermal infection with ds-red expressing* Y. pestis* strain in a GFP-expressing neutrophil transgenic mouse model [137]. The dynamic interactions between ds-red* Y. pestis* and GFP-neutrophils following intradermal infection and the association between* Y. pestis* and neutrophils were confirmed by confocal microscopy which specifically demonstrated that, within 4 hours,* Y. pestis* had been phagocytosed by neutrophils and was intracellular and not just associated with cells. This led the authors to investigate the role of neutrophils in dissemination of plague to the lymph nodes using antibody depletion which suggested that* Y. pestis* subverted the host response very early in infection to prevent dissemination to the lymph nodes.

### 3.3. Biphotonic Imaging: Real-Time* In Vivo* Imaging

Visualising the infectious disease process as it occurs inside a living animal is of major benefit to the development of medical countermeasures. This can be achieved using biphotonic imaging (BPI), a sensitive and noninvasive method of detecting light emitted either as a bioluminescent (BL) or fluorescent (FL) signal, using photon detectors such as those based on a charge coupled device (CCD) camera. BPI has enabled new insights into pathogen dissemination, host responses to infection, interactions between host and pathogen, and the effects of antimicrobials and vaccines. This technique can also be used to refine animal experiments with each animal acting as its own control, therefore, increasing the power of these studies. The creation of BL or FL strains of pathogenic organisms has enabled this field to progress and description of the processes involved in BPI and creation of these strains are comprehensively reviewed in Andreu et al. [138].

Using BPI different patterns of pathogen dissemination can be readily observed allowing discrimination of the growth and spread of different forms of the pathogen or target organs depending on route of infection. For example, BL expressing variants of* B. anthracis* Sterne strain that only produced a BL signal during either spore germination or vegetative growth of the bacterium have permitted identification of sites of germination and spread during the early stages of anthrax infection [139]. BPI studies helped determine the site of anthrax spore germination after inhalational infection [140] where light emitting bacteria in the upper respiratory tract and lung were observed within 30 min of inhalational infection [140, 141].

BPI studies showed that dissemination of* Y. pestis* was found to vary depending on route of infection [142, 143]. Subcutaneous (s.c.) administration in the abdominal* linea alba* region resulted in pathogen spread from the inguinal lymph node (LN) to axillary LN, then to liver and spleen whereas s.c. infection in the more traditional cervical “scruff” region, base of the tail, in the footpad [144] or ear pinna [143] resulted in a different pattern of signal intensity originating at the site of injection. Intranasal (i.n.) challenge with BL* Y. pestis* leads to a detectable BL signal [143, 145] in the upper abdominal region which was confirmed to originate from the lung by* ex vivo* imaging of tissues. BPI has also been used to investigate the dissemination of BL strains of* F. tularensis* type A (SCHU S4) and type B (LVS) [146]. Visualising areas of BL LVS infection and bacterial spread [147] provided important information on the effects of instillation volume and anaesthesia in delivery of i.n. bacterial challenge with the potential to impact on the wider development of* in vivo* pulmonary infection models.

The pattern of dissemination of BL* B. pseudomallei* following inhalational challenge has been investigated [148, 149]. The spread of both wild type and capsule mutant strains of* B. pseudomallei* has been compared in the BALB/c mouse model with an immunocompentent, hairless SKH-1 mouse strain, that is, particularly useful for FL imaging studies due to low autofluorescent background usually generated by fur. Infection studies with a BL strain of* B. mallei* have also been reported [150, 151] with a BL signal detectable in the lung after i.n. challenge from 48 h after infection (p.i.), before progressing to the liver and spleen.

These pathogen dissemination models have subsequently been used to assess the effect of antibiotics and immunotherapies. A substantial reduction of bacterial signal was found in* B. mallei*-infected mice treated with the antibiotic levofloxacin compared to untreated mice [150]; however, when the antibiotic was discontinued at 96 h p.i., reemergence of the BL bacterial signal was observed. This mouse model of* B. mallei* infection has been further used to investigate the effects of CpG treatment alone, previously shown to provide protection against* B. mallei* infection when given preexposure to mice [152]. Mott et al. (2013) [151] used dual signal imaging to elucidate the role of CpGs on neutrophil activation using a neutrophil-specific fluorescent probe. This cyanine 7-conjugate, PEG modified hexapeptide reagent specifically binds to the formylpeptide receptor of neutrophils [136]. This has allowed real-time colocalisation of BL bacterial spread with FL neutrophil responses in the lung during the course of infection.

The effects of immunisation with protective antigen (PA) vaccine demonstrated that, in immunised mice, dissemination of BL* B. anthracis* beyond the nasopharynx region was prevented. The effect of PA vaccine immunisation on anthrax spore germination and bacterial spread has also been assessed [141]. This work clearly demonstrated that although spore germination and bacterial growth occur at the same rate in both immunised and unimmunised mice, bacterial growth was quickly neutralised in the immunised mice whereas BL bacteria spread rapidly in unimmunised controls. BL expressing* B. anthracis* strains modified to express only one of the anthrax toxins stimulated different patterns of early immune response after cutaneous infection of the ear pinna [153]. In this study, draining LN were removed following BPI and immune cell populations were analysed by flow cytometry. The Lethal toxin-expressing strain stimulated increases in the total cell populations of neutrophils, CD4^+^ and CD8^+^ T-cells whereas the immunosuppressive edema toxin-expressing strain stimulated only an increase in CD8^+^ T-cells.

### 3.4. Emerging Technologies

#### 3.4.1. Imaging Flow Cytometry

**Figure 1:**
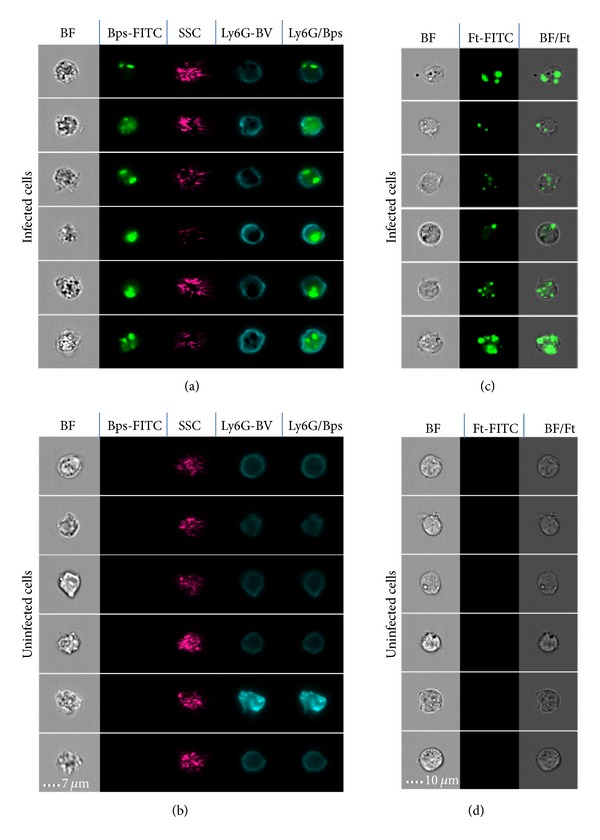
ImageStreamX Mk1 imaging depicting intracellular infection of* ex vivo* and* in vitro* cells. (a) Infected and (b) uninfected* ex vivo* lung cells stained with fluorescently labelled antibodies specific for* Burkholderia pseudomallei* (Bps) and the neutrophil marker Ly6G. A composite (overlayed) fluorescence image shows intracellular Bps inside neutrophils (Ly6G/Bps). Mouse macrophage cell line (P388D.1) infected (c)* in vitro* with* F. tularensis* SCHU S4 (Ft) or uninfected controls (d). Each Image series shows 6 representative cells from one sample. SSC = Side Scatter; BF = Brightfield; FITC = Fluorescein Isothiocyanate; BV = Brilliant Violet.

Recent developments in flow cytometry include the development of imaging flow cytometers including the ImagestreamX. Imaging flow cytometry adds another dimension to flow cytometric applications with images of each cell being produced in addition to fluorescence readouts and has the ability to further advance our understanding of host-pathogen interactions. It is particularly suitable for assessing the colocalisation of pathogens within host cells and for examining cellular processes for uptake and processing of pathogens, for example, phagocytosis, autophagy, and apoptosis. The number of publications which incorporate use of ImagestreamX for investigating host-pathogen interactions is growing each year with a limited number of publications on a wide range of public health related pathogens including* Yersinia enterocolitica* [154],* Plasmodium falciparum* [155, 156], and* Neisseria meningitidis* [157]. As yet, no studies for biodefence pathogens have been published. However, pathogens which have been used to model biodefence pathogens, such as* Yersinia pseudotuberculosis* as a model for* Yersinia pestis*, have been documented [158] demonstrating the potential of this technology. At Defense Science and Technology Laboratory, we are currently using the ImagestreamX to examine the interactions of* B. pseudomallei*,* F. tularensis*, and other pathogens with cellular targets in samples from both* in vivo* and* in vitro* studies (Figure 1). This technique has the potential of providing a high-throughput imaging technique for the analysis of host-pathogen interactions and assessment of immunotherapeutics for biodefence pathogens.

#### 3.4.2. Other Imaging Technologies

Ultrasound magnetic resonance imaging (MRI) and radiography are all technologies which have been used clinically in the diagnosis of infectious diseases including anthrax and tuberculosis. Some of these technologies have been used in biodefence research, for example, positron emission tomography (PET)/computer tomography (CT) imaging was used to examine inflammation patterns in Monkeypox virus infection of primates [159]. The use of these imaging technologies in high containment, alongside optical imaging technologies, has the potential to provide a multi-faceted approach to imaging to further enhance our understanding of the pathogenesis of infection with Biodefence agents in the future. The use of other imaging technologies and their potential application to biodefence disease in the clinic is reviewed in [160].

## 4. Conclusions

Biodefence agents are dangerous pathogens that pose unique challenges for researchers. Human cases of these diseases are relatively rare and therefore animal models play a key role in helping to understand pathogenesis. Imaging and “omic” technologies have greatly aided our ability to study the host response during the course of an infection and have thus provided important insights. Also since it is neither ethical nor feasible to conduct conventional phase III efficacy trials, using biodefence agents in human volunteers, these key technologies can play an important role in the evaluation of vaccines and therapies. They provide evidence to support the concepts defined by the Food and Drug Agency (FDA) Animal Rule [161] for licensing new medical countermeasures.

Currently the majority of studies using the “omic” and imaging techniques, described in this review, have examined the host response independently from pathogen virulence. In the future, however, due to rapid advances in NGS platform technologies and imaging technologies, it is anticipated that examining pathogen virulence whilst simultaneously interrogating host responses will be achieved. Overall, this should reduce and refine animal experiments and thus allow the identification of both host and pathogen markers during infection at the same time. This will further enhance our knowledge of host-pathogen interactions and aid in the development of vaccines and therapeutics for these dangerous pathogens.

## Figures and Tables

**Table 1:** Microarray studies performed with various Biodefence Agents.

Pathogen	Purpose of study	Arrays used	Material tested	Reference
Response to infection				
*Burkholderia pseudomallei *	Profile human antibody responses in healthy and recovered patients.	Protein array containing 154 *B. pseudomallei* proteins.	Human plasma from healthy and recovered melioidosis patients.	[33]
*Burkholderia pseudomallei *	Gene expression changes following intravenous infection with bacteria in BALB/c mice.	Sentrix MouseRef-8 cDNA array (Illumina).	Liver and spleen from BALB/c mice.	[24]
*Burkholderia pseudomallei *	Differences in gene expression after 2 hour exposure to *B. pseudomallei* and *B. thailandensis in vitro*.	GeneChip human genome U133 (Affymetrix).	A549 human lung epithelial cells.	[22]
*Bacillus anthracis *	Gene expression changes in cells exposed to Edema toxin.	GeneChip murine genome (Affymetrix).	RAW 264.7 murine macrophages.	[17]
*Bacillus anthracis *	Gene expression changes in cells exposed to lethal toxin.	GeneChip human genome U133 plus 2.0 (Affymetrix).	Human monocytes from the blood of naïve volunteers.	[8]
*Bacillus anthracis *	Murine macrophage gene expression changes following exposure to protective antigen and lethal factor from *B. anthracis. *	PCR product DNA array.	RAW 264.7 murine macrophages.	[18]
*Bacillus anthracis* spores	Gene expression profiling of human macrophages following infection *in vitro*.	GeneChip human genome U133 Plus 2.0 (Affymetrix).	Human alveolar macrophages following bronchoscopy.	[9]
*Brucella melitensis *	Gene expression analysis of mucosal epithelial cells following infection *in vitro. *	10K human ESTs microarray (Microarray centre, Ontario, Canada).	Epithelial-like human HeLa cell line.	[19]
*Brucella melitensis *	Kinetics of human antibody responses to acute and chronic brucellosis.	*Brucella melitensis* protein array.	Sera from brucellosis patients.	[34]
*Brucella melitensis *	Investigate host gene changes *in vivo* following infection with *Brucella* in a calf ligated ileal loop model.	Custom-made 13K bovine 70 mer oligo array.	Infected Peyer's patch from calf ligated ileal loop.	[31]
*Brucella melitensis *	Full proteome-wide serological analysis of *B. melitensis* in humans.	Protein microarray containing 3046 proteins from *B. melitensis*.	Sera from brucellosis patients.	[35]
*Coxiella burnetii *	Profile humoral immune response of naïve and acute Q-fever patients.	Protein microarray containing 84% of *C. burnetii*.	Human sera from Q-fever patients.	[36]
*Coxiella burnetii *	Comparison of the antibody profiles from acute and chronic Q-fever patients.	Protein microarray containing 93% of *C. burnetii*.	Human sera from Q-fever patients.	[37]
*Coxiella burnetii *	Define the humoral immune profile using Q-fever patient sera.	Custom-made protein microarray containing 19 proteins from *C. burnetii*.	Human sera from Q-fever patients.	[38]
Ebola and Marburg viruses	Gene signatures following infection *in vitro* with Ebola virus, Marburg virus.	Human cDNA array (Agilent).	Human hepatoblastoma (Huh7) cells.	[20]
Ebola virus	Entry into human macrophages. Infection studies *in vitro*.	GeneChip human genome HG-U95Av2 array (Affymetrix).	Primary human macrophages.	[10]
*Francisella tularensis* live vaccine strain (LVS)	Human neutrophil gene expression, *in vitro* infection studies.	GeneChip human genome U133 plus 2.0 (Affymetrix).	Polymorphonuclear leukocytes (PMNs) from human blood.	[11]
*Francisella tularensis* LVS	*In vitro* infection studies using Human PBMCs.	Human gene array (Affymetrix).	Human peripheral blood mononuclear cells (PBMCs).	[12]
*Francisella tularensis* (SchuS4)	Gene expression following inhalation of *F. tularensis* in BALB/c mice.	Mouse array covering 1500 genes. (Ocimumbio).	Lung tissue taken from infected BALB/c mice.	[27]
*Francisella tularensis* (FSC033/snMF)	Gene expression following aerosol exposure with *F. tularensis* in C57BL/6 mice.	Custom-made mouse cDNA array.	Lung tissue taken from infected C57BL/6 mice.	[30]
*Francisella tularensis* (SchuS4)	Gene expression of human monocytes infected *in vitro* with *F. tularensis. *	GeneChip human genome U133 plus 2 (Affymetrix).	Naïve human peripheral blood monocytes.	[13]
*Francisella tularensis* (SchuS4)	Comparison of mouse global transcriptional responses to *F. tularensis, Yersinia pestis, Pseudomonas aeruginosa* and* Legionella pneumophila. *	Mouse whole genome 44K arrays (Agilent).	Lung tissue from infected BALB/c mice.	[29]
Monkeypox and Vaccinia virus	Comparison of gene expression profiles following infection *in vitro* with Monkeypox or Vaccinia virus.	Human cDNA arrays with 406 Variola and Vaccinia virus genes.	Primary human macrophages, primary human fibroblasts and HeLa cells.	[14]
Monkeypox and Vaccinia virus	Comparison of gene expression profiles, *in vitro* infection studies.	Whole human genome oligo microarray (Agilent).	HeLa cells.	[16]
Monkeypox virus	Gene expression changes *in vitro* 3 and 7 hours post-challenge with Monkeypox virus.	Rhesus macaque genome microarrays (Affymetrix).	*Macaca mulatta* kidney cells (MK2).	[21]
Monkeypox virus	Comparison of antibody responses to monkeypox virus infection and human smallpox vaccination.	Protein array covering 92–95% of representative proteins from Monkeypox and Vaccinia virus.	Blood from humans with smallpox vaccination and cyno macaques infected with Monkeypox virus.	[39]
Variola virus	Host gene expression changes in Variola virus infected cynomolgus macaques.	Human cDNA microarrays.	PBMC's sampled from infected monkeys.	[5]
*Yersinia pestis *	Gene expression changes following infection *in vitro. *	Human nylon blots (1185 cDNA spots) (Clontech).	Primary human monocytes and/or mixed with lymphocytes (PBMCs).	[15]
Venezuelan equine encephalitis virus (VEEV)	Gene expression of VEEV infected mice.	Oligo array mouse 70 mer. (Operon) & GEArray, focused mouse Toll-like receptor signaling microarray.	VEEV infected mouse brain CD-1 mice.	[25, 26]

Vaccines				
Anthrax vaccine adjuvant CpG ODN	Measure gene expression changes in mice and splenocytes treated with CpG ODN.	Murine oligonucleotide array (custom-made).	Spleens and splenocytes from various breds of mice.	[40–42]
*Francisella tularensis* LVS	Assess the memory response of PBMCs taken from LVS vaccinated and naïve humans.	GeneChip human genome U133 (Affymetrix).	Re-stimulated PBMCs from LVS vaccinated and naïve humans.	[43]
Killed *Francisella tularensis *(LVS) adjuvanted with ISCOMS admixed with CpG	Define antibody profiles of vaccinated mice.	Whole proteome microarray custom-made.	BALB/c mice vaccinated with LVS.	[44]
Q-fever vaccine	Assess antibody immune profiles of Q-Vax vaccinated humans.	*C. burnetii *protein microarray (custom-made).	Sera from vaccinated humans.	[45]
Smallpox vaccines	Assess antibody profiles generated to MVA, Acam2000 and/or Dryvax smallpox vaccines.	Protein array containing Vaccinia virus proteins [46].	Mouse, rabbit, macaque, black-tailed prairie dog and human sera.	[47–51]

Therapies				
Anti-coagulant treatments for Ebola virus infection	Comparison of NHP host genome responses responding to candidate therapeutics following infection with Ebola virus.	Human genome cDNA microarray.	PBMC's from rhesus macaques infected with ZEBOV and treated shortly after exposure with rNAPc2 or rhAPC.	[32]

Omission from this table does not constitute absence of data.
